# Reduction of flavonoid content in honeysuckle via *Erysiphe lonicerae*-mediated inhibition of three essential genes in flavonoid biosynthesis pathways

**DOI:** 10.3389/fpls.2024.1381368

**Published:** 2024-04-16

**Authors:** Mian Zhang, Jie Zhang, Qiaoqiao Xiao, Yulong Li, Shanshan Jiang

**Affiliations:** ^1^Guizhou University of Traditional Chinese Medicine, Guiyang, China; ^2^College of Life Sciences, Shaanxi Normal University, Xi’an, China

**Keywords:** *Lonicera japonica*, *Erysiphe lonicerae*, flavonoid, chlorogenic acid, medicinal ingredient

## Abstract

Honeysuckle, valued for its wide-ranging uses in medicine, cuisine, and aesthetics, faces a significant challenge in cultivation due to powdery mildew, primarily caused by the *Erysiphe lonicerae* pathogen. The interaction between honeysuckle and *E. lonicerae*, especially concerning disease progression, remains insufficiently understood. Our study, conducted in three different locations, found that honeysuckle naturally infected with *E. lonicerae* showed notable decreases in total flavonoid content, with reductions of 34.7%, 53.5%, and 53.8% observed in each respective site. Controlled experiments supported these findings, indicating that artificial inoculation with *E. lonicerae* led to a 20.9% reduction in flavonoid levels over 21 days, worsening to a 54.8% decrease by day 42. Additionally, there was a significant drop in the plant’s total antioxidant capacity, reaching an 81.7% reduction 56 days after inoculation. Metabolomic analysis also revealed substantial reductions in essential medicinal components such as chlorogenic acid, luteolin, quercetin, isoquercetin, and rutin. Investigating gene expression revealed a marked decrease in the relative expression of the *LjPAL1* gene, starting as early as day 7 post-inoculation and falling to a minimal level (fold change = 0.29) by day 35. This trend was mirrored by a consistent reduction in phenylalanine ammonia-lyase activity in honeysuckle through the entire process, which decreased by 72.3% by day 56. Further analysis showed significant and sustained repression of downstream genes *LjFNHO1* and *LjFNGT1*, closely linked to *LjPAL1*. We identified the mechanism by which *E. lonicerae* inhibits this pathway and suggest that *E. lonicerae* may strategically weaken the honeysuckle’s disease resistance by targeting key biosynthetic pathways, thereby facilitating further pathogen invasion. Based on our findings, we recommend two primary strategies: first, monitoring medicinal constituent levels in honeysuckle from *E. lonicerae*-affected areas to ensure its therapeutic effectiveness; and second, emphasizing early prevention and control measures against honeysuckle powdery mildew due to the persistent decline in crucial active compounds.

## Introduction

Honeysuckle (*Lonicera japonica*), a plant with a history of over 1,500 years in Chinese herbal medicine, was first mentioned in Hongjing Tao’s “Ming Yi Bie Lu” (Supplementary Records of Famous Physicians) during the Wei and Jin dynasties (220-450 AD). It has since been a staple in major Chinese pharmacopoeias, including the current version (Chinese Pharmacopoeia Commission, 2020). In Traditional Chinese Medicine, honeysuckle is valued for its ‘cold’ properties, believed to be effective against ‘hot’ ailments. Besides its medicinal uses, fresh honeysuckle is also utilized in making healthful teas, as well as for garnishing and flavoring in culinary arts, and is a popular choice in traditional Chinese gardens and art for its aesthetic and olfactory appeal (Zheng et al., 2022). Recent scientific studies have highlighted its extensive potential clinical benefits, encompassing antibacterial, antiviral, antifungal, anti-inflammatory, antioxidative, antipyretic, antitumor, and anti-chronic disease effects (Chang and Hsu, 1992; Yu et al., 2008; Chen et al., 2013; Jiang et al., 2014; Kwon et al., 2015; Li et al., 2015; Zhou et al., 2015; Li et al., 2018; Wang et al., 2019; Li et al., 2024). Its notable antiviral properties have gained significant attention, particularly for its inhibitory effects on various animal viruses and human influenza, leading to increased research and development during the Covid-19 pandemic (Huang et al., 2023; Shi et al., 2024; Zhang et al., 2023a). Honeysuckle is predominantly found in East Asia, especially in China, Japan, and Korea, with a notable concentration in southern China (Li et al., 2024). The cultivation and trade of honeysuckle, particularly its dried flower buds, form a significant market in China, generating over 2 billion yuan annually and showing consistent growth (Shang et al., 2011). The estimated global market value of honeysuckle in 2022 was approximately USD 322 million. It is projected to expand to an estimated USD 494 million by the year 2030. As one of the most commonly used herbs in East Asia, honeysuckle remains a key subject in scientific research. Recent advancements, such as the sequencing of its genome and the development of platforms for functional gene analysis, have propelled the study of honeysuckle into the realm of molecular research (Pu et al., 2020; Xiao et al., 2021).

Investigations into the key active components that contribute to the therapeutic efficacy of honeysuckle have emerged as a significant area of scientific inquiry (Pan et al., 2020). To date, over 600 secondary metabolites have been identified in honeysuckle, including diverse species such as volatile oils, cyclic ether terpenes, flavonoids, phenolic acids, triterpenoids, saponins, and various alkaloids (Yang et al., 2021; Tan et al., 2023; Li et al., 2024). Flavonoids, a notable class of these secondary metabolites, are produced by plants through a prolonged evolutionary process characterized by synergistic interactions with environmental factors and pathogens, aiding in the plant’s defense against adversities and diseases. These flavonoids, when consumed in certain herbal medicines, are directly assimilated by humans, and extensive research has validated their range of positive physiological impacts on human and animal health, highlighting their essential role in the medicinal properties of these plants (Ramaroson et al., 2022; Roy et al., 2022). The Chinese Pharmacopoeia of 2020 stipulates a minimum flavonoid content of 0.05% in high-quality honeysuckle herbs, underscoring the significance of flavonoids as a critical quality control metric (Chinese Pharmacopoeia Commission, 2020). A thorough review of existing literature on honeysuckle flavonoids has revealed the presence of 62 unique flavonoids, with documented evidence of their multifaceted functionalities including antibacterial, antiviral, anti-tumor, antioxidant, anti-inflammatory, and analgesic properties. Furthermore, these flavonoids contribute to liver and gallbladder health, lipid regulation, immune system modulation, cough relief, and allergy treatment (Ren et al., 2008; Zheng et al., 2022; Li et al., 2024). These findings are in strong agreement with the traditional medicinal applications of honeysuckle in treating a variety of ailments, thereby emphasizing the pivotal role of flavonoids in its therapeutic potency. The ongoing research into flavonoids continues to be a focal point in the broader context of honeysuckle research.

Powdery mildew (PM), a widespread plant disease caused by fungi from the Erysiphales order within the Ascomycota phylum, significantly affects a variety of plant species globally (Hacquard, 2014). Characterized as predominantly biotrophic pathogens, Erysiphales do not immediately kill their host plants; instead, they infect and develop a nutrient-absorbing structure known as a ‘haustorium’ (Kuhn et al., 2016). This pathology has profound implications for global agriculture and economic sectors. The *in vitro* cultivation of these biotrophic pathogens presents a considerable challenge, impeding research and leading to a dearth of effective treatment methods (Sirangelo, 2023). Honeysuckle, a plant not immune to this affliction, is particularly susceptible to *E. lonicerae*, a pathogen responsible for significant damage (Pigul and Dmitrieva, 2013). The rapid spread of this pathogen, affecting leaves, flowers, and stems, leads to diminished yields and consequential economic losses (Rakhimova et al., 2005). Despite being a common disease in large-scale honeysuckle cultivation, research on powdery mildew in honeysuckle lags behind that of major food crops, largely because honeysuckle is not categorized among traditional bulk food crops. Furthermore, as a medicinal plant, honeysuckle is subject to stricter pesticide usage regulations, complicating the management and control of its powdery mildew disease. Consequently, current research on honeysuckle powdery mildew predominantly concentrates on initial stages of plant disease study, such as the screening of agents for field prevention and control (He et al., 2022) and the identification and phylogenetic analysis of the pathogen (Choi et al., 2023; He and Bai, 2023). However, there remains a paucity of knowledge regarding the interaction mechanisms between honeysuckle and its powdery mildew pathogen *E. lonicerae*, as well as the specific physiological and biochemical impacts of *E. lonicerae* infection on honeysuckle.

In the present study, our objective is to delineate the impacts of *E. lonicerae* infection on the biosynthesis and accumulation of flavonoids in honeysuckle. We aim to thoroughly investigate the underlying molecular pathways and mechanisms that are influenced by *E. lonicerae*. Additionally, this research intends to assess the extent to which the medicinal properties of honeysuckle are compromised due to the infection, and based on these findings, propose appropriate recommendations to mitigate such damage. This comprehensive analysis will provide deeper insights into the interaction between honeysuckle and the pathogen, contributing significantly to the development of more effective strategies for preserving the medicinal integrity of this important plant.

## Materials and methods

### Plant materials

*L. japonica* plants were cultivated in the medicinal plant garden at Guizhou University of Chinese Medicine, located in Guiyang, China. This site is positioned at 26.38° N latitude and 106.59° E longitude, with an elevation averaging 1100 meters. The region experiences an average maximum temperature of 25°C and an average minimum temperature of 16°C, contributing to favorable growing conditions for these plants. The honeysuckle utilized in this experiment were all five-year-old plants.

### Inoculation of *E. lonicerae* on honeysuckle

The inoculation and sampling of honeysuckle were carried out from July to September 2022 in the medicinal plant garden at Guizhou University of Chinese Medicine. Spores were meticulously harvested from infected honeysuckle leaves, a process which involved the delicate transfer of visible white spores from the leaf surface into a sterile centrifuge tube containing 1.5 mL of sterile water. This initial spore suspension was then diluted tenfold with sterile water. The spore density in the resultant solution, measured in spores per milliliter, was determined using a hemocytometer. Subsequently, the spore concentration was precisely adjusted to a target density of 3.0×10^5^ spores/mL by adding a calculated volume of sterile water. This adjustment in concentration was verified through additional hemocytometric assessments. For inoculation, this spore suspension was evenly sprayed onto healthy honeysuckle leaves, utilizing approximately 50 mL of suspension per plant. In the case of the control group, an equivalent volume of sterile water was applied. The experiment was conducted with 20 pots for each treatment, ensuring one plant per pot. The Disease Severity Index (DSI) was calculated employing the formula: DSI = [Sum (Disease Grade × Number of Infected Leaves)]/(9 × Total Number of Leaves) × 100, as delineated by He et al. (2022).

### Detection of enzyme activity of phenylalanine ammonia-lyase

Phenylalanine ammonia-lyase (PAL) activities were evaluated utilizing the Phenylalanine Deaminase Activity Assay Kit, procured from Beijing Solarbio Biotechnology Co., Ltd., Beijing, China. The assays were conducted in strict accordance with the protocols specified in the kit’s instructions, employing the pre-prepared reagents included within the kit. Flower bud samples were collected, promptly immersed in liquid nitrogen to ensure rapid freezing, subsequently ground into a fine powder, and thereafter, the prescribed quantity of each sample, as delineated in the kit’s instructions, was utilized for the assay.

### Detection of total flavonoids

Field sampling for the present study was carried out in Xiaoguan Township, Zunyi City, Guizhou Province, in June 2022. Within this region, three non-contiguous honeysuckle fields, each exhibiting symptoms of powdery mildew, were randomly selected for the research. In every field, 20 healthy plants and 20 plants infected with powdery mildew were chosen at random for analytical purposes. The flavonoid content in each infected and uninfected plant across the fields was subsequently measured. For each field, the average flavonoid content was calculated separately for the uninfected and infected plants. The study proceeded to compare these averages to evaluate the disparity in flavonoid content between the infected and uninfected plants in each of the three chosen fields. Flavonoid quantification was executed utilizing the aluminum chloride colorimetric method, as delineated by Chang et al. (2002). This analysis was enabled through the use of the Plant Flavonoids Assay Kit supplied by Solarbio Life Sciences, China.

### Total antioxidant capacity assay and the detection of H_2_O_2_ concentration

Flower buds of honeysuckle were pulverized using liquid nitrogen and subsequently sieved to produce a fine powder suitable for various assays. The preparation of water extracts from each plant part was conducted employing ultrasound-assisted extraction (UAE) at ambient temperature. This procedure entailed the application of 200 watts of ultrasound for a duration of 20 minutes. The total antioxidant capacity (T-AOC) of these extracts was ascertained utilizing the T-AOC assay kit (BC1315, Solarbio, Beijing, China). This assay relies on the reduction of tripyridyltriazine-Fe^3+^ (TPTZ-Fe^3+^) to tripyridyltriazine-Fe^2+^ (TPTZ-Fe^2+^) under acidic conditions, an indicator of the sample’s total antioxidant capacity. Absorbance readings at 593 nm were recorded using a microplate reader (Thermo Fisher Scientific, Waltham, MA, USA). Additionally, hydrogen peroxide (H_2_O_2_) concentrations in the samples were quantified using specific detection assay kits (Solarbio, Beijing, China). These kits are based on the principle that H_2_O_2_ reacts with titanium sulfate, forming a yellow titanium peroxide complex. This complex exhibits a distinct absorbance at 415 nm, which is instrumental in quantifying the H_2_O_2_ content in the samples.

### Methods and procedures of qPCR analysis

In this research, we focused on examining the relative expression of key genes involved in flavonoid biosynthesis. To achieve this, first strand cDNA was synthesized using the Revert Aid™ First Strand cDNA Synthesis Kit (Fermentas, Shenzhen, China), with strict adherence to the manufacturer’s instructions. The expression levels of these genes were quantitatively analyzed through Quantitative Polymerase Chain Reaction (qPCR), with each reaction performed in quintuplicate. For these assays, TB Green Premix Ex Taq, provided by Takara Biomedical Technology, was utilized. In this context, *LjActin* was employed as the internal control gene. The relative expression of the targeted genes was calculated using the 2^-ΔΔCt^ method, which is based on the cycle threshold (Ct) values. Detailed information about the gene accession numbers and the specific primers used for these experiments is extensively documented in the Supporting Information, particularly in **Supplementary Table S4**.

### Sample collection and preparation for LC-MS/MS

Fresh honeysuckle flower bud tissues, weighing 80 mg, were harvested on the 42nd day following inoculation. These samples were promptly snap-frozen in liquid nitrogen and subsequently ground into a fine powder using a mortar and pestle. For the purposes of homogenization and metabolite extraction, 1000 μL of methanol/acetonitrile/H_2_O mixture in a 2:2:1 volume/volume/volume (v/v/v) ratio was utilized. This mixture was then centrifuged for 20 minutes at 14,000 g at a temperature of 4°C. Post-centrifugation, the supernatant was meticulously collected and dried employing a vacuum centrifuge. For the Liquid Chromatography-Mass Spectrometry (LC-MS) analysis, the dried extract was re-dissolved in 100 μL of an acetonitrile/water solution (1:1, v/v). This solution underwent an additional round of centrifugation for 15 minutes at 14,000 rpm and 4°C. The supernatant obtained from this final centrifugation step was then utilized for injection in the LC-MS analysis.

### LC-MS/MS analysis

Analyses were performed using a UHPLC system (1290 Infinity LC, Agilent Technologies) in conjunction with a quadrupole time-of-flight mass spectrometer (AB Sciex TripleTOF 6600) at Shanghai Applied Protein Technology Co., Ltd. The prepared samples, each represented by six biological replicates, were separated using an Agilent 1290 Infinity LC ultra-high-performance liquid chromatography (UHPLC) HILIC column, maintained at 40 °C. The flow rate was set at 0.4 ml/min, with an injection volume of 2 μL. The chromatographic mobile phase comprised two components: Mobile phase A, containing 25 mM ammonium acetate and 0.5% formic acid in water, and Mobile phase B, consisting of methanol. A gradient elution protocol was established, starting with 5% phase B for the initial 0.5 minutes, linearly increasing to 100% B over the next 9.5 minutes (0.5-10 min). This 100% B composition was then maintained from 10 to 12 minutes. Subsequently, from 12.0 to 12.1 minutes, the composition was rapidly reduced from 100% back to 5% B, which was maintained for the final 3.9 minutes (12.1-16 min) of the run. During the procedure, samples were stored at 4°C in an automatic injector, and random sequencing of sample analysis was employed to minimize instrument-related variations. Quality control (QC) samples were interspersed throughout the run to ensure data consistency and accuracy.

The ESI source parameters for mass spectrometry were as follows: Gas1 and Gas2 at 60, curtain gas (CUR) at 30, with the source temperature held at 600°C. The IonSpray Voltage Floating (ISVF) was set to ± 5500 V. In the MS-only acquisition mode, the instrument recorded data in the mass-to-charge ratio (m/z) range of 60-1000 Da, with a TOF MS scan accumulation time of 0.20 s/spectrum. For auto MS/MS acquisition, data was captured in the m/z range of 25-1000 Da, with a product ion scan accumulation time of 0.05 s/spectrum. The product ion scan operated on information-dependent acquisition (IDA) in high sensitivity mode. The parameters included a fixed collision energy (CE) of 35 V with a ± 15 eV tolerance and declustering potential (DP) set to 60 V (+) and -60 V (-), while excluding isotopes within 4 Da. A maximum of 10 candidate ions were selected for monitoring in each cycle.

### Data process

The processed data, following normalization to total peak intensity, underwent analysis utilizing the “ropls” package in R, which implements sophisticated multivariate data analysis techniques. This analysis encompassed Pareto-scaled Principal Component Analysis (PCA) and Orthogonal Partial Least-Squares Discriminant Analysis (OPLS-DA). The reliability of the model was ascertained through a 7-fold cross-validation and response permutation testing, ensuring the validity and robustness of the model. Within the OPLS-DA framework, Variable Importance in the Projection (VIP) values were calculated for each variable, shedding light on their individual contributions to the model’s classification accuracy. Metabolites with VIP values exceeding 1.0 underwent additional analysis via a univariate Student’s t-test, aimed at determining the statistical significance of their contributions. Metabolites presenting p-values less than 0.05 were deemed statistically significant, highlighting a discernible disparity in their concentrations between the groups under comparison. For Kyoto Encyclopedia of Genes and Genomes (KEGG) pathway analysis, the KEGG database (https://www.kegg.jp/) was utilized for a comprehensive examination of omics data. Fisher’s Exact Test was applied in the KEGG pathway enrichment analysis, evaluating the overrepresentation of metabolites within specific pathways. This assessment also considered the involvement of either the species under investigation or species closely related. The significance of observed variations in metabolic pathways was determined by P-values, with lower values indicating greater statistical significance. This methodological approach facilitated a thorough understanding of metabolic alterations and their possible biological implications.

### Statistical analysis

In our statistical analysis conducted with IBM SPSS 22.0 software, we analyzed differences between uninfected and infected groups using Student’s t-test (α = 0.05). For datasets involving more than two groups, we applied Analysis of Variance (ANOVA) and Duncan’s Multiple Range Test for pairwise comparisons, both at a significance level of α = 0.05.

## Results

### Significant reduction in honeysuckle’s total flavonoid content due to natural *E. lonicerae* infection

**Figure 1:**
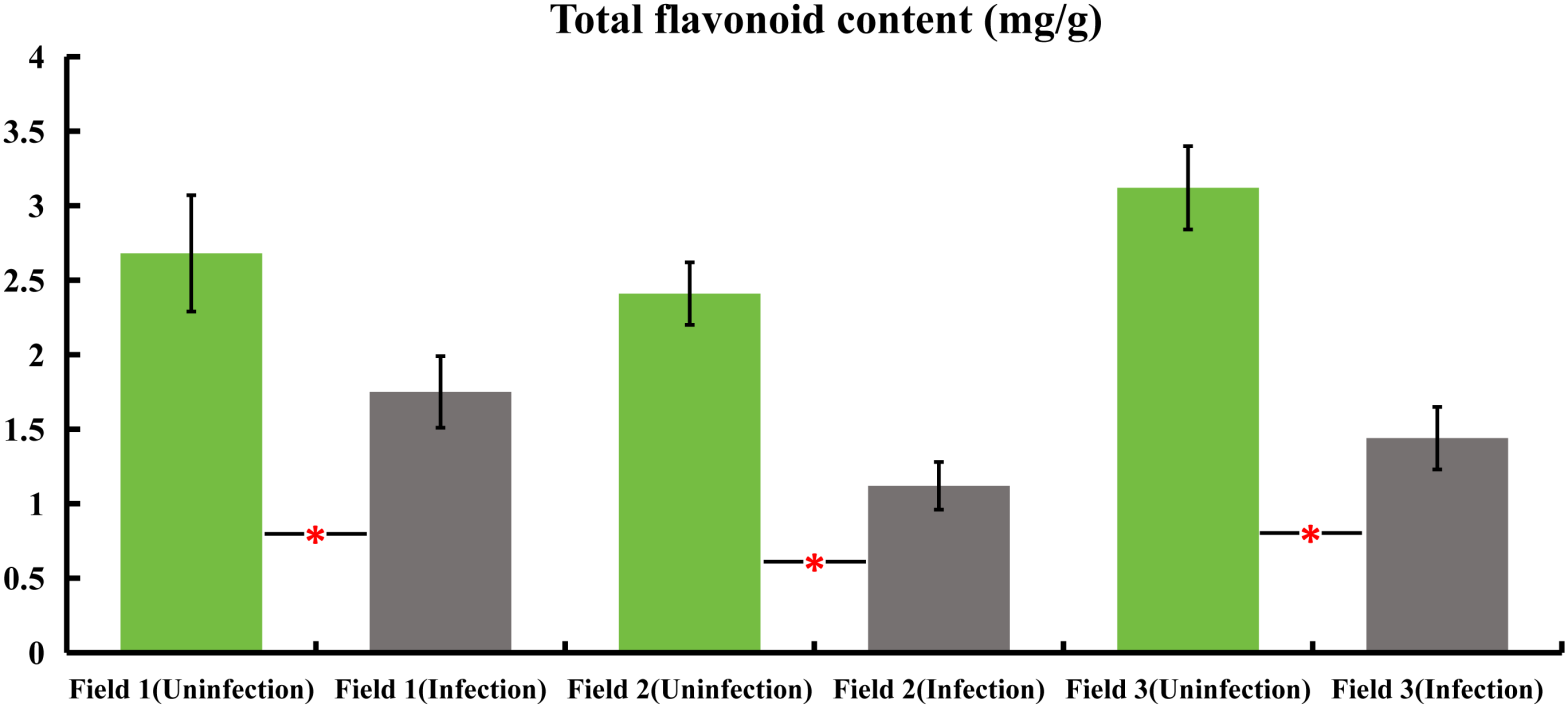
variations in total flavonoid content in honeysuckle across three distinct plots, comparing infected and non-infected samples with powdery mildew. All the data were showed as mean ± SD (n = 20). Statistical significance is indicated by asterisks (P < 0.05).

Samples were collected from honeysuckle plants naturally infected with powdery mildew, as well as from healthy plants, across three distinct honeysuckle plantation plots. These samples were analyzed to determine the total flavonoid content. The findings revealed a significant decrease in the total flavonoid content of the infected plants compared to the healthy ones. Specifically, in field 1, the total flavonoids in infected plants were reduced by 34.7%, field 2 showed a decrease of 53.5%, and field 3 exhibited a reduction of 53.8% in infected plants as compared to their non-infected counterparts. These results indicate a substantial reduction in total flavonoid content in honeysuckle plants when naturally infected with powdery mildew (**Figure 1**).

### Progressive decline in total flavonoid content of honeysuckle post-inoculation with *E. lonicerae*

**Figure 2:**
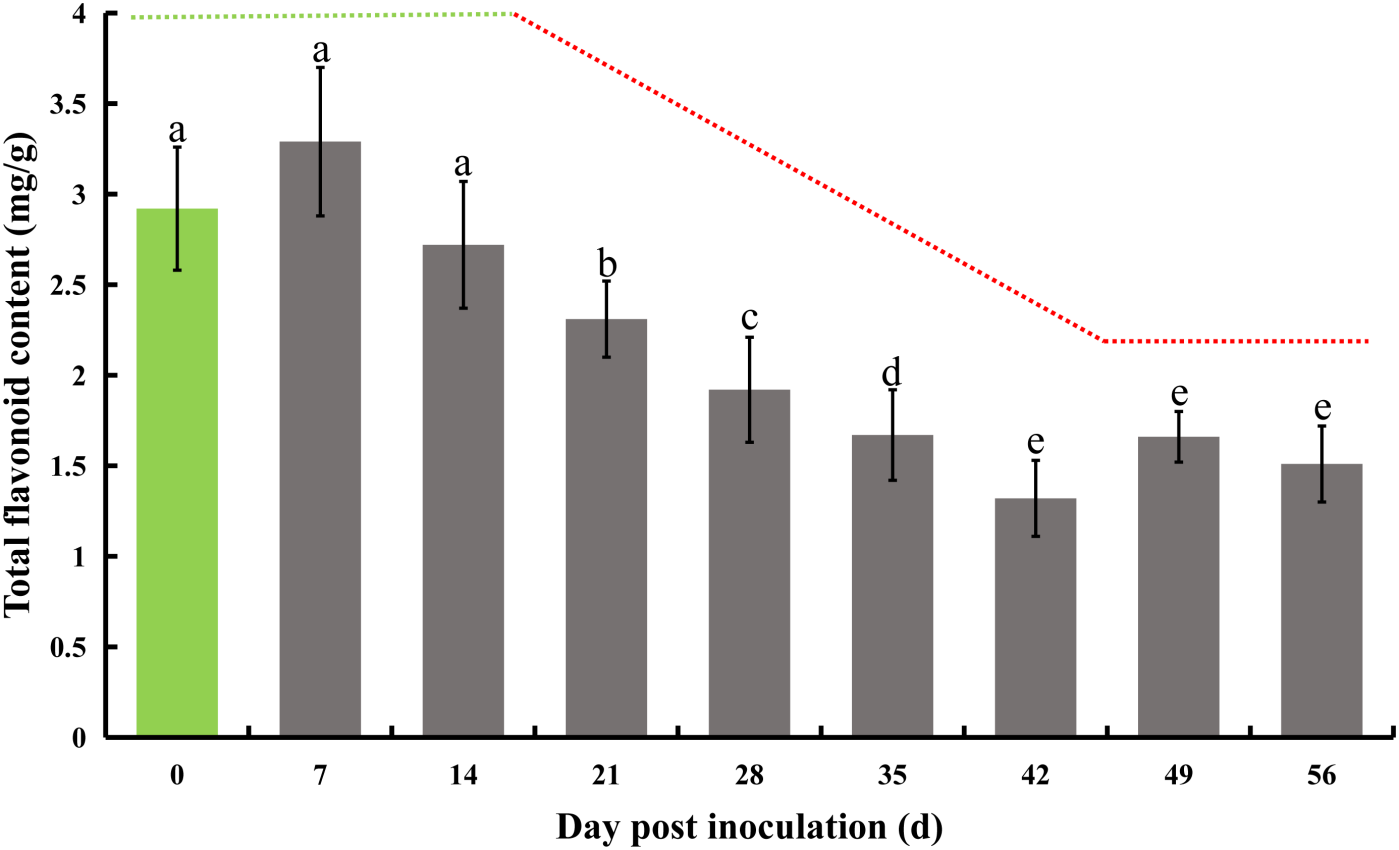
Total flavonoid content in honeysuckle post-inoculation with *E. lonicerae* over an 8-week period. The inoculation day served as the control (Day 0). A marked and consistent decrease in total flavonoids was observed, beginning from the 21st day post-inoculation. The data were showed as mean ± SD (n = 5). Statistical significance are indicated by different letters (P < 0.05).

The initial total flavonoid test was conducted immediately following inoculation, serving as a baseline control on day 0, and subsequently repeated every 7 days. A notable decline of 20.9% in total flavonoid content was first observed on day 21, with this significant decrease continuing until day 42, culminating in a 54.8% reduction. A consistent and marked downward trend was observed between days 14 and 42. Specifically, there was a 15.1% decrease in total flavone on day 21 compared to day 14. On day 28, the total flavonoids decreased by 16.9% relative to day 21, followed by a 13.0% decrease on day 35 compared to day 28, and a 21.0% decrease on day 42 compared to day 35. Beyond this, there were no further significant reductions on days 49 and 56, although the total flavone content remained significantly lower than initial levels (**Figure 2**).

### Continuous decrease in total antioxidant capacity of honeysuckle following *E. lonicerae* inoculation

**Figure 3:**
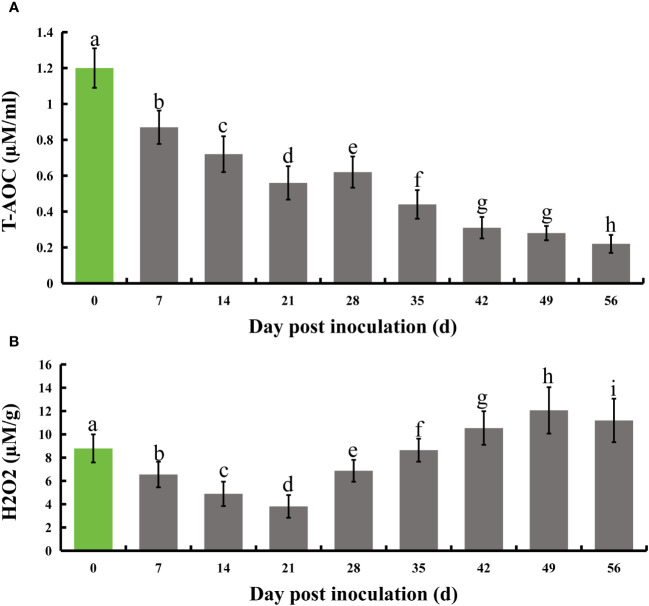
Evolution of total antioxidant capacity and H_2_O_2_ levels in honeysuckle over an 8-week period following *E lonicerae* inoculation. Day 0, the day of inoculation, was established as the control point. **(A)** Demonstrates a significant and continuous decline in total antioxidant capacity commencing from the 7th day post-inoculation. **(B)** Illustrates a significant reduction in H_2_O_2_ content starting from the 7th day post-inoculation, followed by a notable increase on the 28th day. The data were showed as mean ± SD (n = 5). Statistical significance are indicated by different letters (P < 0.05).

Following infection of honeysuckle with *E. lonicerae*, we monitored its total antioxidant capacity (T-AOC) at seven-day intervals for eight weeks. The results indicated a significant decrease in T-AOC starting from the first measurement on day 7 post-inoculation, continuing through the subsequent seven measurements over two months. Relative to the control, there was a 27.5% decrease in T-AOC on day 7, 40% on day 14, 53.3% on day 21, 48.3% on day 28, 63.3% on day 35, 74.2% on day 42, 76.7% on day 49, and 81.7% on day 56. Concurrently, we assessed the internal hydrogen peroxide (H_2_O_2_) levels using the same testing frequency. The results showed a notable initial decrease in H_2_O_2_ levels post-inoculation, followed by a significant increase. Specifically, H_2_O_2_ levels decreased by 25.5%, 44.4%, 56.7%, and 21.8% on days 7, 14, 21, and 28, respectively. On day 35, the H_2_O_2_ levels were not significantly different from the control. However, there was a substantial increase by 16.6%, 37.2%, and 27.3% on days 42, 49, and 56, respectively, compared to the control (**Figure 3**).

### Metabolomic differences in flavonoid metabolism between *E. lonicerae* infected and uninfected honeysuckle

**Figure 4:**
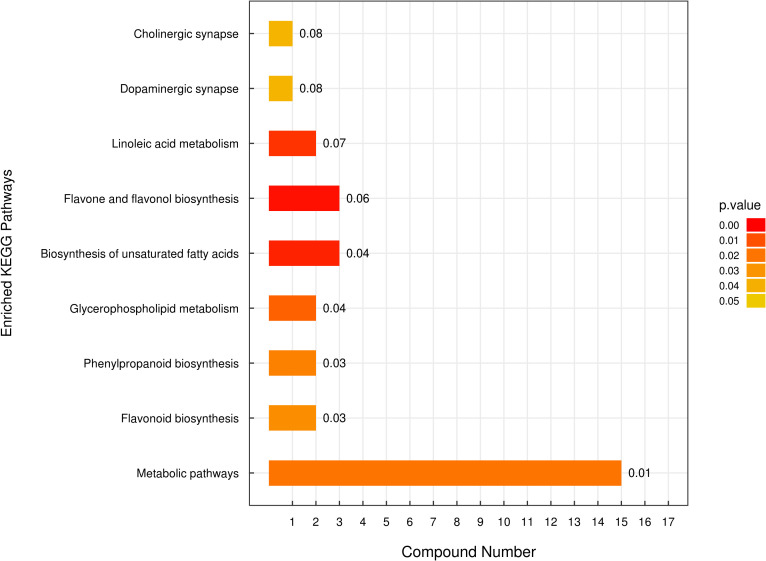
Enriched Kyoto Encyclopedia of Genes and Genomes (KEGG) Pathways Analysis. The vertical axis displays distinct KEGG metabolic pathways, while the horizontal axis quantifies the number of Differentially Expressed Metabolites (DEMs) associated with each pathway.

Metabolomics analysis was utilized to identify differences in secondary metabolites between *E. lonicerae*-inoculated (42nd day) and uninoculated (0th day) honeysuckle plants. This analysis detected 736 secondary metabolites across 13 superclasses (**Supplementary Figures S1**, **S3**; **Supplementary Table S1**). Of these, 80 were identified as significantly differentially expressed secondary metabolites (**Supplementary Figure S2**; **Supplementary Table S2**). Subsequent Kyoto Encyclopedia of Genes and Genomes (KEGG) analysis of these differentially expressed metabolites (DEMs) highlighted notable differences in the lipid and flavonoid synthesis pathways between healthy, uninfected honeysuckle and those infected with *E. lonicerae* (**Figure 4**).

The metabolome analysis also revealed that six flavonoids were significantly more abundant in healthy, uninfected honeysuckle compared to the infected plants. These included quercetin (Fold change > 15), hispidulin (Fold change > 4), isoquercitrin (Fold change > 1.5), rutin (Fold change > 1.5), and luteolin (Fold change > 1.5). Notably, chlorogenic acid, identified as an effective medicinal component and a quality indicator in honeysuckle, showed a greater than 3-fold increase in the uninfected plants compared to the infected ones (**Supplementary Table S3**).

### *E. lonicerae* suppression of flavonoid and chlorogenic acid synthesis related gene (*LjPAL1*) and total phenylalanine ammonia-lyase activity in honeysuckle

**Figure 5:**
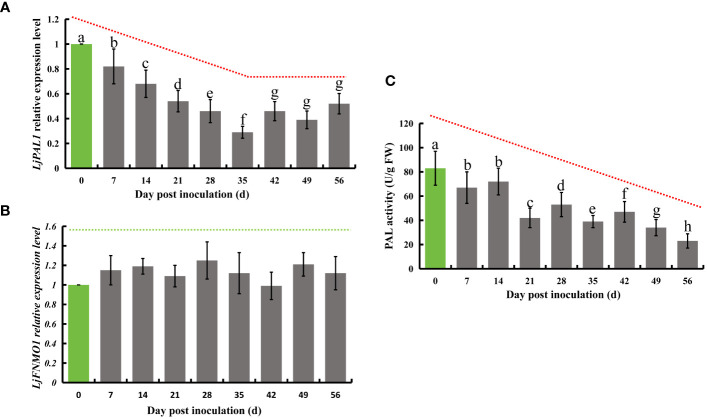
Relative expression of *LjPAL1* and *LjFNMO1* genes and detection of phenylalanine deaminase enzyme activity in honeysuckle post-inoculation with *E lonicerae* over an 8-week period, with day 0 as the control. **(A)** Demonstrates a significant, continuous down-regulation in *LjPAL1* gene expression starting from the 7th day post-inoculation. **(C)** Highlights a significant decrease in phenylalanine aminotransferase activity beginning on the 7th day post-inoculation. **(B)** Indicates no significant changes in *LjFNMO1* gene expression following inoculation. The data were showed as mean ± SD (n = 5). Statistical significance are indicated by different letters (P < 0.05).

Phenylalanine is known to undergo a series of transformations mediated by phenylalanine deaminase, eventually leading to the production of chlorogenic acid. This compound is further processed by quinate monooxygenase to partly form luteolin. In honeysuckle, *LjPAL1* has been identified as a gene encoding phenylalanine deaminase, while *LjGNMO1* encodes quinate monooxygenase. Upon inoculation with *E. lonicerae*, the expression of the *LjPAL1* gene in honeysuckle showed a significant decline, beginning from the 7th day post-inoculation (Fold change = 0.82). This decrease in gene expression continued, reaching its lowest level on the 35th day (Fold change = 0.29), and remained significantly reduced (Fold change < 0.6) until the end of the study period. Concurrent with these observations, the enzyme activity of total PALs was also significantly reduced from day 7 onwards, continuing to decrease until day 56. The reductions were 19.3%, 13.3%, 49.4%, 36.1%, 53.0%, 43.4%, 59.0%, and 72.3% across the eight weekly assessments. In contrast, no significant expression differences were detected for the *LjGNMO1* gene throughout the study period (**Figure 5**).

### Inhibition of *LjFNHO1* and *LJFNGT1* by *E. lonicerae* affects synthesis of flavonoids like quercetin, lignoceroside, and rutin in honeysuckle

**Figure 6:**
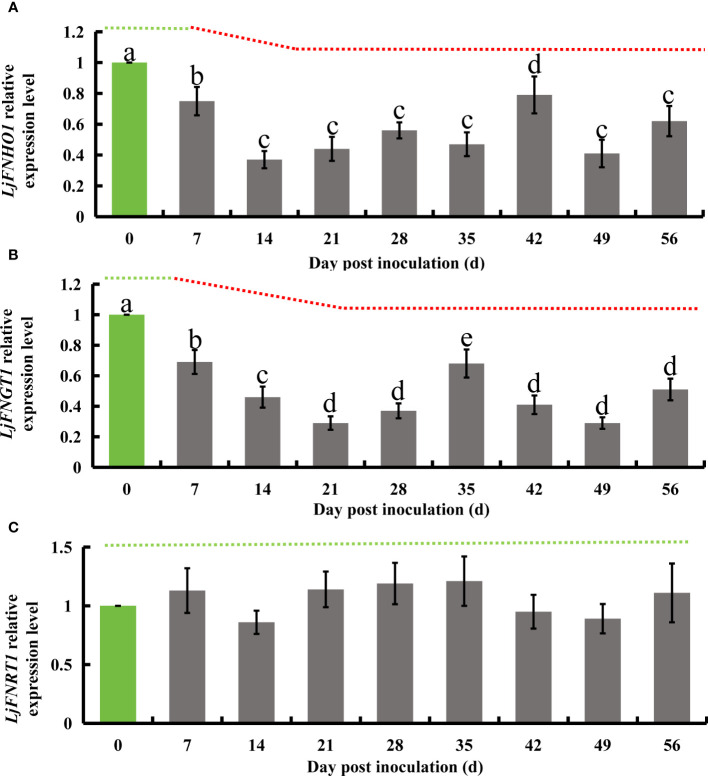
Relative gene expression alterations in honeysuckle following *E lonicerae* inoculation over an 8-week period, using day 0 as control. **(A)** Shows a significant, continuous down-regulation in *LjFNHO1* gene expression commencing from the 7th day post-inoculation. **(B)** Presents a consistent, significant down-regulation in *LjFNGT1* gene expression starting from the 7th day post-inoculation. **(C)** Reveals no significant changes in *LjFNRT1* gene expression. The data were showed as mean ± SD (n = 5). Statistical significance are indicated by different letters (P < 0.05).

Flavonoid hydroxylase, an enzyme in the flavonoid biosynthesis pathway, catalyzes the conversion of kaempferol into quercetin. Subsequently, flavonol glucosyltransferase acts on quercetin to produce isoquercetin, which is then transformed into rutin by flavonol L-rhamnosyltransferase. Additionally, flavonoid hydroxylase can directly synthesize luteolin from apigenin. In honeysuckle, the homologous genes corresponding to these enzymes have been identified: *LjFNHO1* for flavonoid hydroxylase, *LjFNGT1* for flavonol glucosyltransferase, and *LjFNRT1* for flavonol L-rhamnosyltransferase. Following successful inoculation with *E. lonicerae*, quantitative PCR (qPCR) analysis conducted weekly over 8 weeks revealed significant regulatory changes in these genes. The expression of *LjFNHO1* was significantly down-regulated starting from the 7th day (Fold change = 0.75), with a sharp decrease noted on the 14th day (Fold change = 0.37). This reduced expression level persisted (Fold change < 0.8) throughout the remainder of the study. The *LjFNGT1* gene, which encodes flavonol glucosyltransferase, exhibited a significant decline in expression level on the 7th day (Fold change = 0.69), further decreasing on the 14th day (Fold change = 0.46) and the 21st day (Fold change = 0.29). From the 28th day onwards, the expression of *LjFNGT1* remained significantly lower (Fold change < 0.7). In contrast, the relative expression level of *LjFNRT1*, the gene for flavonol L-rhamnosyltransferase, did not exhibit significant downregulation throughout the duration of the detection process (**Figure 6**).

### *E. lonicerae*’s role in down-regulating *LjPAL1*, *LjFNHO1*, and *LjFNGT1* to diminish chlorogenic acid, rutin, luteolin, quercetin, and isoquercetin synthesis

**Figure 7:**
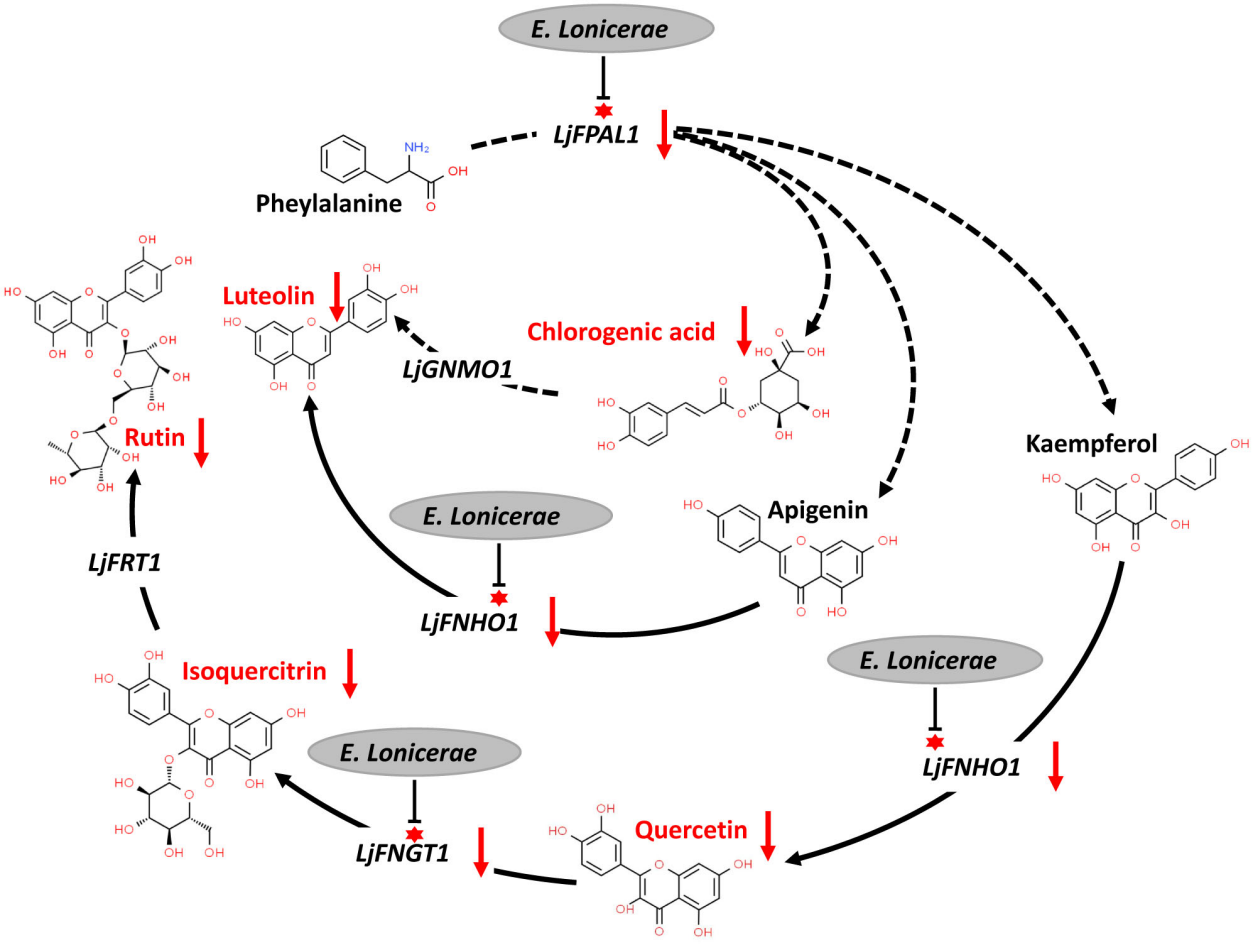
inhibition of flavonoid synthesis pathway in honeysuckle by *E. lonicerae*. This figure illustrates the suppression of *LjPAL1*, *LjFNHO1*, and *LjFNGT1* genes in the pathway by *E. lonicerae* infection, leading to a significant decrease in the corresponding flavonoids.

The interaction between *E. lonicerae* and honeysuckle remains underexplored. Based on the observed gene expression changes in honeysuckle and the accumulation of secondary metabolites following infection with *E. lonicerae*, we have delineated the mechanism by which *E. lonicerae* inhibits flavonoid synthesis in honeysuckle. This mechanism can be divided into three distinct pathways (**Figure 7**). In the first pathway, *E. lonicerae* persistently suppresses the expression of the *LjPAL1* gene, which is an upstream gene in the pathway. This inhibition leads to a reduction in chlorogenic acid content, contributing to the decreased luteolin levels. Concurrently, in the second pathway, *E. lonicerae* down-regulates the expression of *LjFNHO1*, a crucial gene for converting apigenin into luteolin. This down-regulation represents another factor contributing to the reduced luteolin content. In the third pathway, the suppression of *LjFNHO1* gene expression by *E. lonicerae* results in diminished quercetin synthesis. The reduced quercetin levels, along with the inhibition of *LjFNGT1* which is another important gene in this pathway, collectively lead to a decrease in isoquercitrin production. This, in turn, further impedes the synthesis of rutin. Thus, the cumulative effect of these three pathways underlies the reduced flavonoid synthesis in honeysuckle post *E. lonicerae* infection (**Figure 7**).

## Discussion

### The strategic inhibition of flavonoid accumulation: a potential infection tactic of *E. lonicerae*

Flavonoids and their precursors are pivotal substances in plants, synthesized as a response to biotic or abiotic stresses. These compounds play an essential role in plant defense, functioning as signaling molecules or phytoalexins (Ramaroson et al., 2022; Zhao et al., 2024). Their significance is particularly notable in safeguarding plants against various microorganisms, including phytopathogenic bacteria (Kröner et al., 2012; Hijaz et al., 2013; Li et al., 2022), fungi (Lu et al., 2017; Calla, 2022; Zhang et al., 2023b), and viruses (Krcatović et al., 2008; Yao et al., 2019; Yuan et al., 2019; Ramaroson et al., 2022). Recent research has further illuminated the role of plant flavonoids in enhancing pathogen resistance. This enhancement is achieved through the modulation of rhizosphere microorganisms (Wu et al., 2023), indicating an evolving understanding of the complex interactions within the plant microbiome and its impact on plant health. Flavonoids represent a critical barrier that microbes encounter during their interactions with plants. As biochemical gatekeepers, these compounds play a vital role in determining the nature and extent of microbial activity in the plant environment. Their presence often dictates whether a plant-microbe interaction will be beneficial, neutral, or harmful to the plant, thus significantly influencing the plant’s overall health and resilience.

Recent research has increasingly shown in various economically significant plants that flavonoids play an integral role in plant resistance to powdery mildew, positioning them as key substances in combating this disease (Yu et al., 2022; Xu et al., 2023). When *E. lonicerae* invades honeysuckle, it initially confronts the direct or indirect defense mechanisms presented by these abundant flavonoids. This interaction presents a co-evolutionary challenge for *E. lonicerae*, particularly as a biotrophic pathogen seeking long-term survival within its host. Consequently, circumventing flavonoid synthesis becomes a critical strategy for the pathogen’s success. Our experimental observations revealed a significant reduction in flavonoid content in honeysuckle, both in natural infections and following artificial inoculations with *E. lonicerae*. Notably, this decline in total flavonoid content appears to weaken the plant’s resistance, with a marked decrease observed as early as the 14th day post-infection. This timing coincides with a rapid spread of the pathogen and a significant increase in the disease severity index (as illustrated in **Supplementary Figure S4**). These findings suggest that *E. lonicerae* may actively suppress flavonoid levels to enhance its infectivity.

### Direct inhibition of flavonoid synthesis by *E. lonicerae*: a cost-effective strategy against honeysuckle flavonoids

Direct targeting and depletion of flavonoids by pathogens would be an inefficient and resource-intensive approach. Instead, inhibiting flavonoid synthesis at its origin emerges as a more strategic method. Post-inoculation of honeysuckle with *E. lonicerae*, we observed a consistent decrease in the expression of the phenylalanine deaminase gene (*LjPAL1*) and a reduction in total PAL enzyme activity, which are pivotal in flavonoid and precursor synthesis (Cochrane et al., 2004; Chen et al., 2023). Furthermore, significant down-regulation was noted in the flavonoid hydroxylase gene *LjFNHO1* and flavonol glucosyltransferase gene *LjFNGT1*, both integral to downstream flavonoid biosynthesis (Shimada et al., 1999; Kubo et al., 2007; Chen et al., 2023; Negi et al., 2023). Post-inoculation, a decrease in downstream flavonoids such as chlorogenic acid, luteolin, quercetin, isoquercetin, and rutin was detected. Investigating the mechanisms through which the pathogen inhibits critical genes in flavonoid synthesis presents an intriguing research avenue, potentially pivotal for identifying disease resistance breeding targets and enhancing the screening of honeysuckle genetic resources.

Additionally, we assessed the levels of H_2_O_2_, which plays a complex role in flavonoid synthesis in plants, acting beneficially in moderation but detrimentally in excess (Saxena et al., 2016; Zibanezhadian et al., 2020; Wei et al., 2024). A marked decrease in H_2_O_2_ levels was observed during the initial 7-21 days post-infection, followed by a significant rise, exceeding levels in uninfected plants after day 42. This pattern indicates that *E. lonicerae* may initially suppress H_2_O_2_ in honeysuckle to avert additional defense activation, then later exploits it to sustain parasitism. The intricate relationship between flavonoids and H_2_O_2_, where flavonoids can either promote or inhibit H_2_O_2_ synthesis depending on the context (Yokomizo and Moriwaki, 2006; Wei et al., 2024), suggests that *E. lonicerae* is attempting to strike a balance that benefits both its infestation and subsequent parasitism by modulating flavonoid synthesis.

Moreover, we propose the hypothesis that *E. lonicerae* may deploy effector proteins to disrupt key genes in the honeysuckle flavonoid synthesis pathway. Effectors are known to be potent tools for plant pathogens (Kotsaridis et al., 2023; Sabnam et al., 2023; Oliveira-Garcia et al., 2024). The future discovery of such an effector, adept at regulating host flavonoids and related redox reactions *in vivo*, would be highly significant.

### *E. lonicerae*’s impact beyond yield: direct reduction of medicinal properties and value in honeysuckle

Historically, plant disease research, particularly in economically significant crops such as food staples and large fruits, has predominantly focused on yield impact. Yield, the most conventional and straightforward indicator of agricultural productivity, has long been the gold standard for assessing the extent of disease damage and the effectiveness of control methods (Large, 1966; Chiarappa et al., 1972; Gaunt, 1995; Koenning and Wrather, 2010; Mehmood et al., 2023; Sajitha et al., 2024). However, in the case of medicinal plants like honeysuckle, which do not fall into the categories of food or fruit, the scope of concern should broaden beyond yield. The impact of disease on the medicinal value, particularly the potency of key active components, is equally paramount. A diminution in the concentration of these active ingredients can lead to a substantial decrease in both the economic value and practical utility of the medicinal plant, even if the total yield is unaffected. Our study on honeysuckle powdery mildew underscores this essential consideration.

Flavonoids are acknowledged as pivotal medicinal components in plants, renowned for their wide array of therapeutic properties including anti-inflammatory, anti-microbial, anti-tumor, antioxidant, and anti-viral effects, particularly in the context of various chronic diseases (Panche et al., 2016; Tungmunnithum et al., 2018; Chen et al., 2023). Moreover, they serve as key quality markers for honeysuckle, with the 2020 Chinese Pharmacopoeia specifying a minimum content threshold of 0.05% for the herb to be considered medicinally qualified (Zheng et al., 2022). Specific flavonoids like luteolin, quercetin, isoquercetin, and rutin have been recognized for their potent antibacterial, anti-inflammatory, antioxidant, and antitumor properties, which are fundamental to the therapeutic efficacy of medicinal plants (Seelinger et al., 2008; Naveed et al., 2018; Zwicker et al., 2019; Yang et al., 2020; Zheng et al., 2024). Chlorogenic acid and luteolin, in particular, are abundant in honeysuckle and have garnered extensive pharmacological interest due to their efficacy as medicinal components (Wang, 2017; Liu et al., 2023). Chlorogenic acid, alongside total flavonoid content, is also employed as a quality control benchmark in honeysuckle, as evidenced by the 2020 Chinese Pharmacopoeia, which mandates a minimum content of 1.5% (Zheng et al., 2022). Consequently, any reduction in either total flavonoid content or the concentrations of these specific active compounds directly impinges upon the economic and medicinal value of honeysuckle.

Unfortunately, our research has uncovered a significant and persistent reduction in both the total flavonoid content and specific active compounds in honeysuckle following infection by *E. lonicerae*. This finding implies that the efficacy of honeysuckle per unit weight, whether for the isolation and purification of these substances or for direct use in herbal medicine, is likely diminished. Given this impact, it becomes crucial to vigilantly monitor the levels of active ingredients in honeysuckle, especially in powdery mildew-prone regions and at the critical juncture of harvest. Additionally, our study reveals that after the onset of powdery mildew, there is a continuous decline in both flavonoids and several other active ingredients in honeysuckle, with no signs of recuperation observed during the two-month duration of the study. This underscores the importance of early intervention and control measures for powdery mildew to preserve the medicinal integrity of the honeysuckle.

### Honeysuckle powdery mildew research: a field marked by challenges and promising opportunities

Powdery mildew represents a significant challenge for a wide range of human-cultivated plant species. This disease, which has a global distribution, affects nearly all common crops and is a major concern within the plant pathology community due to the severe economic losses it causes annually worldwide (Lebeda et al., 2024; Lu et al., 2024; Stack et al., 2024; Xue et al., 2024). Moreover, research on powdery mildew in certain key crops and model plants has progressed to the stage of investigating immune mechanisms at a molecular level (Wu et al., 2024). Honeysuckle is not immune to powdery mildew, a disease recognized as one of the most devastating to its cultivation, particularly in large-scale production. The disease has led to significant yield losses (Glawe, 2008; Pigul and Dmitrieva, 2013; Hacquard, 2014; Cui and He, 2022). However, research on honeysuckle powdery mildew remains nascent. To date, investigations have primarily focused on identifying control agents, quantifying economic impacts, and conducting pathogen phylogenetic analyses (He et al., 2013; Bradshaw et al., 2021; He et al., 2022; Choi et al., 2023; He and Bai, 2023). There is a notable gap in our understanding of the specific molecular disruptions that occur following infection of honeysuckle by *E. lonicerae*. Our knowledge about this disease remains limited, with few in-depth studies available to comprehensively understand its impact, particularly on the medicinal value of honeysuckle, where the concentration of key active ingredients is as critical as yield. The recent publication of honeysuckle’s genomic information (Pu et al., 2020), the development of biomics analysis tools (Xiao et al., 2021), the emerging understanding of the microbiological characteristics of *E. lonicerae*, and improvements in *in vivo* honeysuckle inoculation techniques (He et al., 2022) all provide a conducive foundation for advancing research on honeysuckle powdery mildew. With these developments, the study of this disease is poised to enter a new and promising phase.

## Conclusion

Throughout its invasion and proliferation, *E. lonicerae* actively inhibits the expression of three critical genes in honeysuckle – *LjPAL1*, *LjFNHO1*, and *LjFNGT1* – which are integral to the plant’s flavonoid biosynthesis pathway. This inhibition results in a significant reduction in the flavonoid content, thereby markedly disrupting the redox equilibrium within the host plant. This observed pattern underscores the flavonoid synthesis pathway as a key site of interaction between *E. lonicerae* and honeysuckle, suggesting that a major vulnerability in the plant’s immune defense against the pathogen may reside here. The potential for natural mutations within these down-regulated genes to confer enhanced resistance to powdery mildew presents a compelling avenue for further research. Our findings lay a strong groundwork for the exploration of honeysuckle germplasm for resistance to powdery mildew and provide guidance for future efforts in disease resistance breeding. Furthermore, the prospect that *E. lonicerae* may employ specific effectors to target this pathway opens up intriguing possibilities for future investigations.

In contrast to food crops, the management of diseases in medicinal plants like honeysuckle should extend beyond yield considerations to encompass the impact of disease on essential medicinal components. This study highlights that powdery mildew not only poses a threat in terms of economic losses but also has the potential to compromise the therapeutic efficacy and medicinal value of infected honeysuckle. Consequently, this underscores the necessity for stringent quality control in honeysuckle fields afflicted with powdery mildew, particularly focusing on these critical medicinal components. Given the observed continual and exacerbating decline in these components, the early implementation of preventive and control measures against powdery mildew becomes increasingly advantageous. Based on these findings, there is a strong impetus for the establishment of a new, more systematic and integrated approach to the prevention, control, and monitoring of honeysuckle powdery mildew.

## Data availability statement

The datasets presented in this study can be found in online repositories. The names of the repository/repositories and accession number(s) can be found in the article/**Supplementary Material**.

## Author contributions

MZ: Conceptualization, Data curation, Methodology, Writing – original draft, Writing – review & editing, Funding acquisition, Investigation, Project administration, Resources. JZ: Conceptualization, Data curation, Methodology, Writing – original draft, Writing – review & editing, Supervision, Validation. QX: Funding acquisition, Investigation, Methodology, Resources, Writing – review & editing. YL: Data curation, Investigation, Software, Validation, Writing – review & editing. SJ: Writing – review & editing, Methodology, Investigation, Software, Visualization.

## Supplementary material

Supplementary Figure 1 Comprehensive metabolite profiling in the study. This figure categorizes all detected metabolites, their superclass affiliations, and their respective proportions.

Supplementary Figure 2 Identification of 736 secondary metabolites from 13 families. This analysis, conducted through positive and negative Electrospray Ionization (ESI) modes, identified 80 significantly differing secondary metabolites. **(A)** Depicts DEMs detected in positive ESI modes. **(B)** Illustrates DEMs detected in negative ESI modes.

Supplementary Figure 3 Principal Component Analysis (PCA) results with 7-fold cross-validation. **(A)** Displays the PCA model parameter R_2_X (cumulative) = 0.646 under the positive ESI model. **(B)** Shows the PCA model parameter R_2_X (cumulative) = 0.793 under the negative ESI model. Green represents the infected group, blue denotes the uninfected group, and purple signifies the quality control group.

Supplementary Figure 4 Disease severity index (DSI) of honeysuckle after inoculation with *E. lonicerae*. After inoculation, the DSI of Honeysuckle continued to increase significantly from 14.2 at day 7 to 76.9 at day 56. The data were showed as mean ± SD (n = 5). Statistical significance are indicated by different letters (P < 0.05).

Supplementary Table 1 Comprehensive profile of secondary metabolites and metabolome analysis. This table provides detailed information on all secondary metabolites identified in this experiment.

Supplementary Table 2 Comparative analysis of secondary metabolites in honeysuckle: Infected vs Uninfected. Detailed data on differential secondary metabolites between *E. lonicerae*-infected and uninfected honeysuckle samples are presented.

Supplementary Table 3 Enriched differential pathways and associated dems in kegg analysis. This table offers in-depth information on significantly enriched differential pathways identified in the KEGG analysis, including the DEMs involved in these pathways.

Supplementary Table 4 Primers utilized in qpcr analysis. A detailed list of all primers used for quantitative PCR (qPCR) analysis in this study is provided.
