# Supplementary figures and images for: Reduction of flavonoid content in honeysuckle via Erysiphe lonicerae-mediated inhibition of three essential genes in flavonoid biosynthesis pathways
Source: Front Plant Sci. 2024 Apr 16;15:1381368. doi: 10.3389/fpls.2024.1381368 (PMC11059088; doi:10.3389/fpls.2024.1381368)

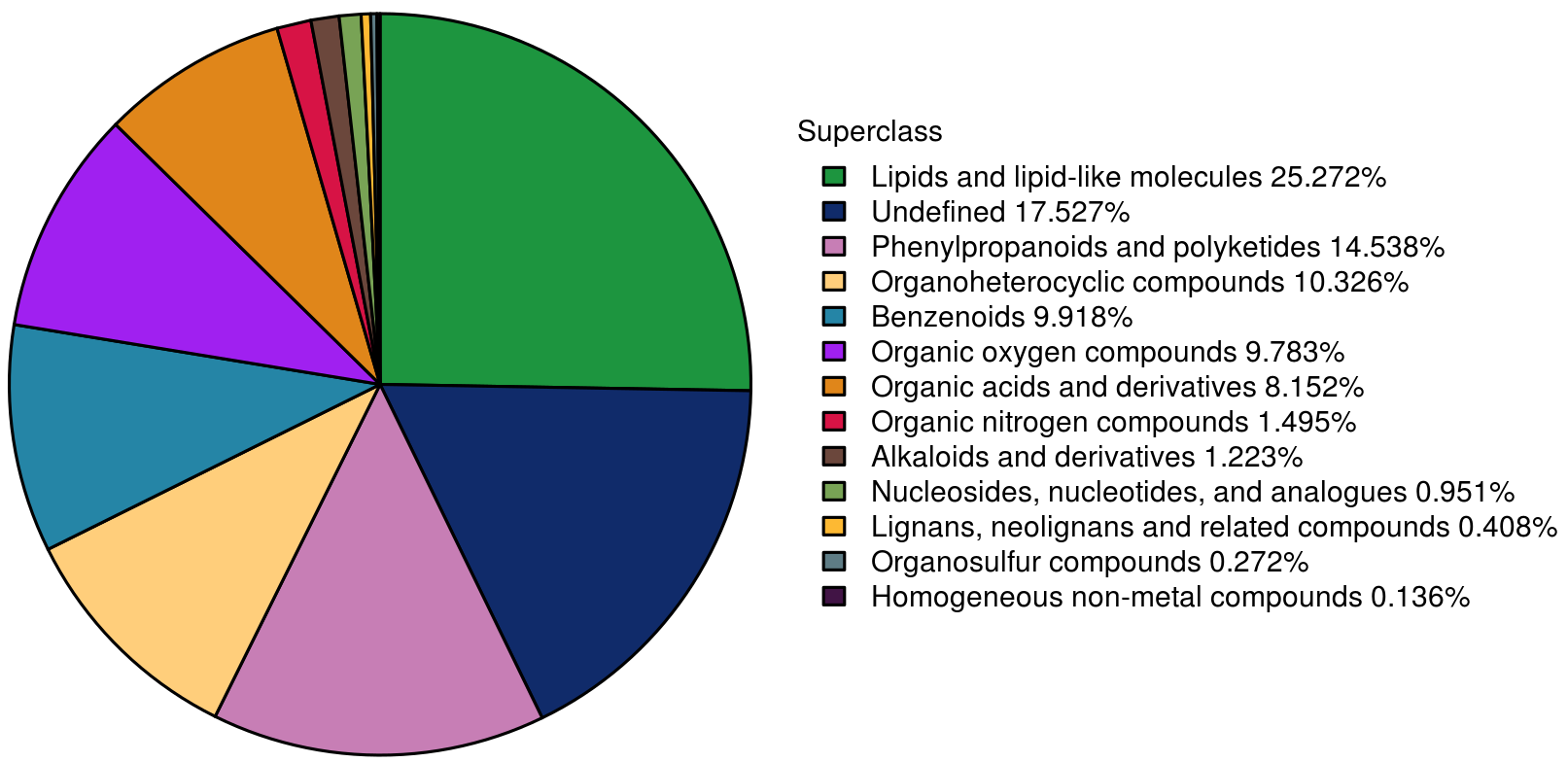

Supplement: Supplementary Figure 1 — Comprehensive metabolite profiling in the study. This figure categorizes all detected metabolites, their superclass affiliations, and their respective proportions. [file Image_1.tif]

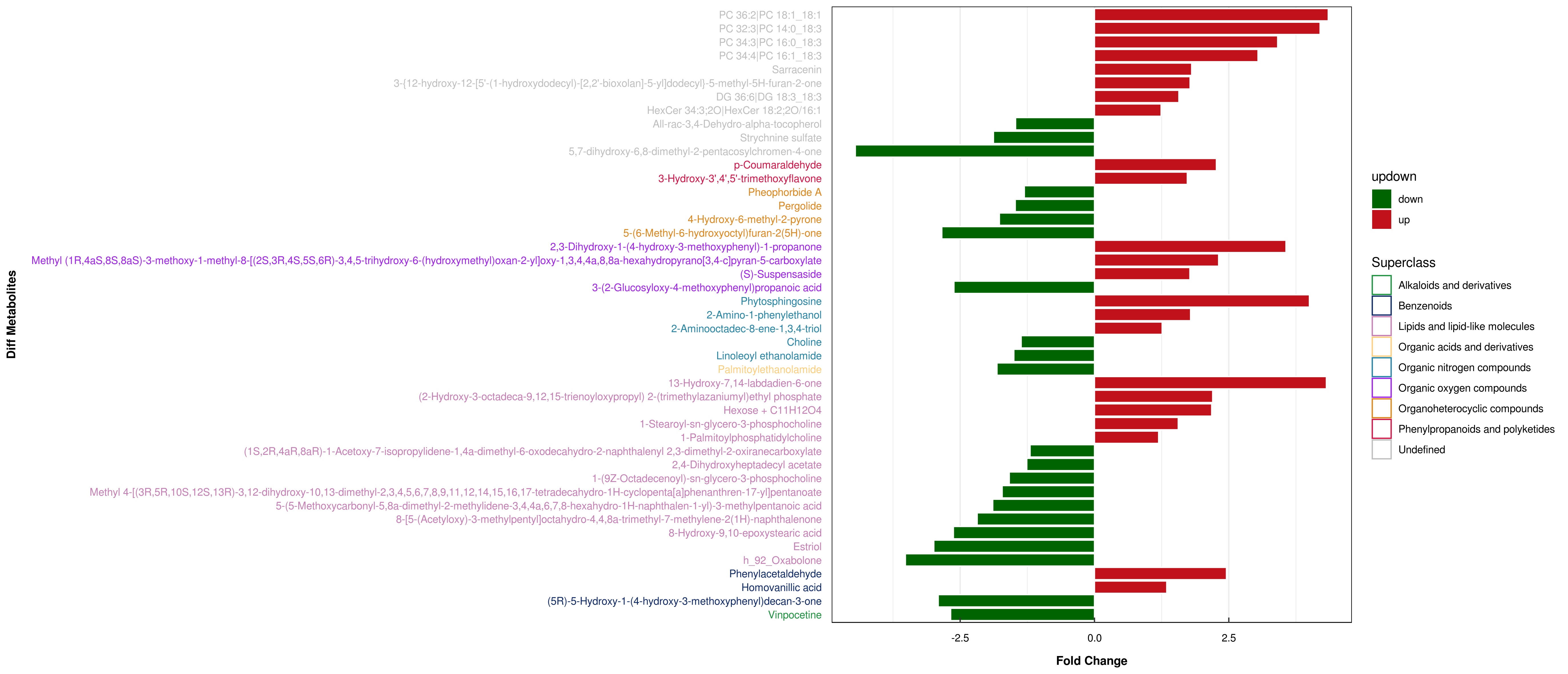

Supplement: Supplementary Figure 2 — Identification of 736 secondary metabolites from 13 families. This analysis, conducted through positive and negative Electrospray Ionization (ESI) modes, identified 80 significantly differing secondary metabolites. (A) Depicts DEMs detected in positive ESI modes. (B) Illustrates DEMs detected in negative ESI modes. [file Image_2.tif]

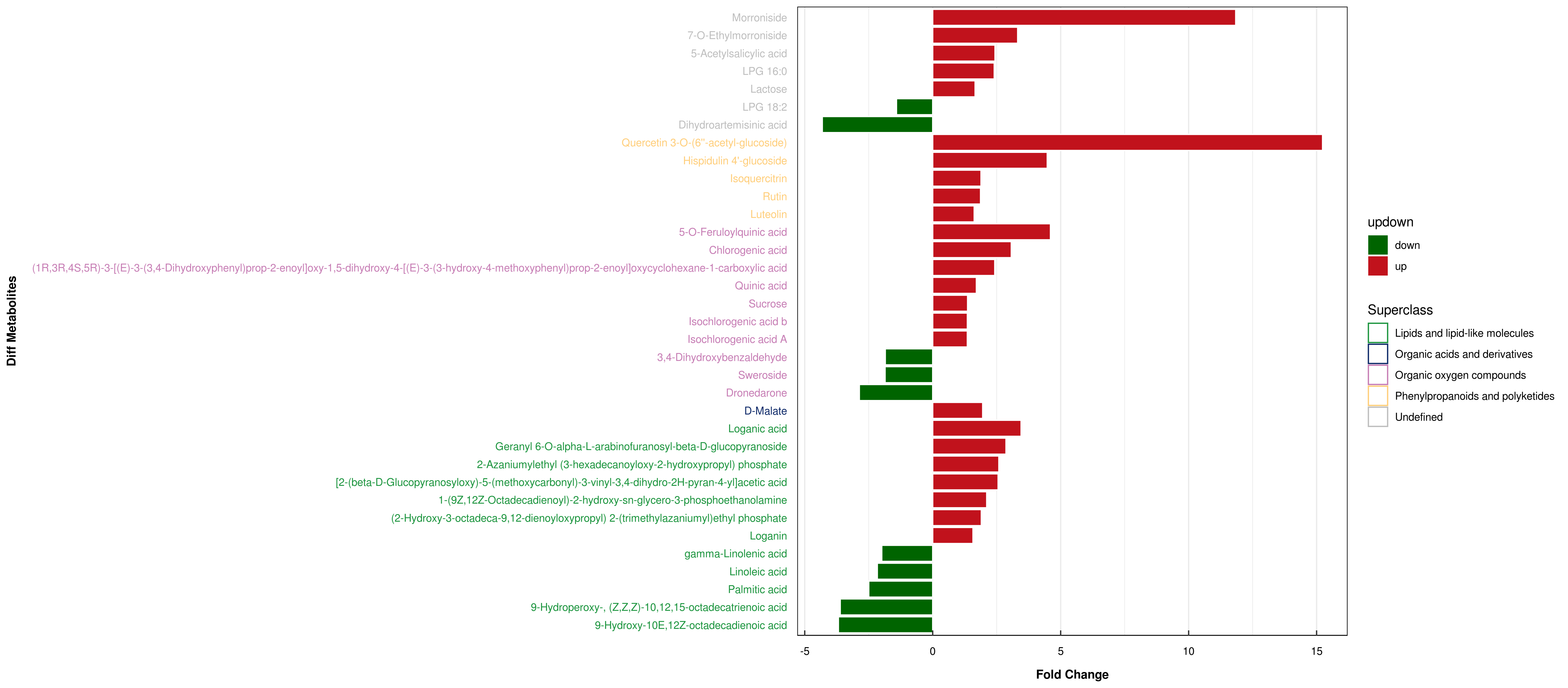

Supplement: Supplementary Figure 3 — Principal Component Analysis (PCA) results with 7-fold cross-validation. (A) Displays the PCA model parameter R2X (cumulative) = 0.646 under the positive ESI model. (B) Shows the PCA model parameter R2X (cumulative) = 0.793 under the negative ESI model. Green represents the infected group, blue denotes the uninfected group, and purple signifies the quality control group. [file Image_3.tif]

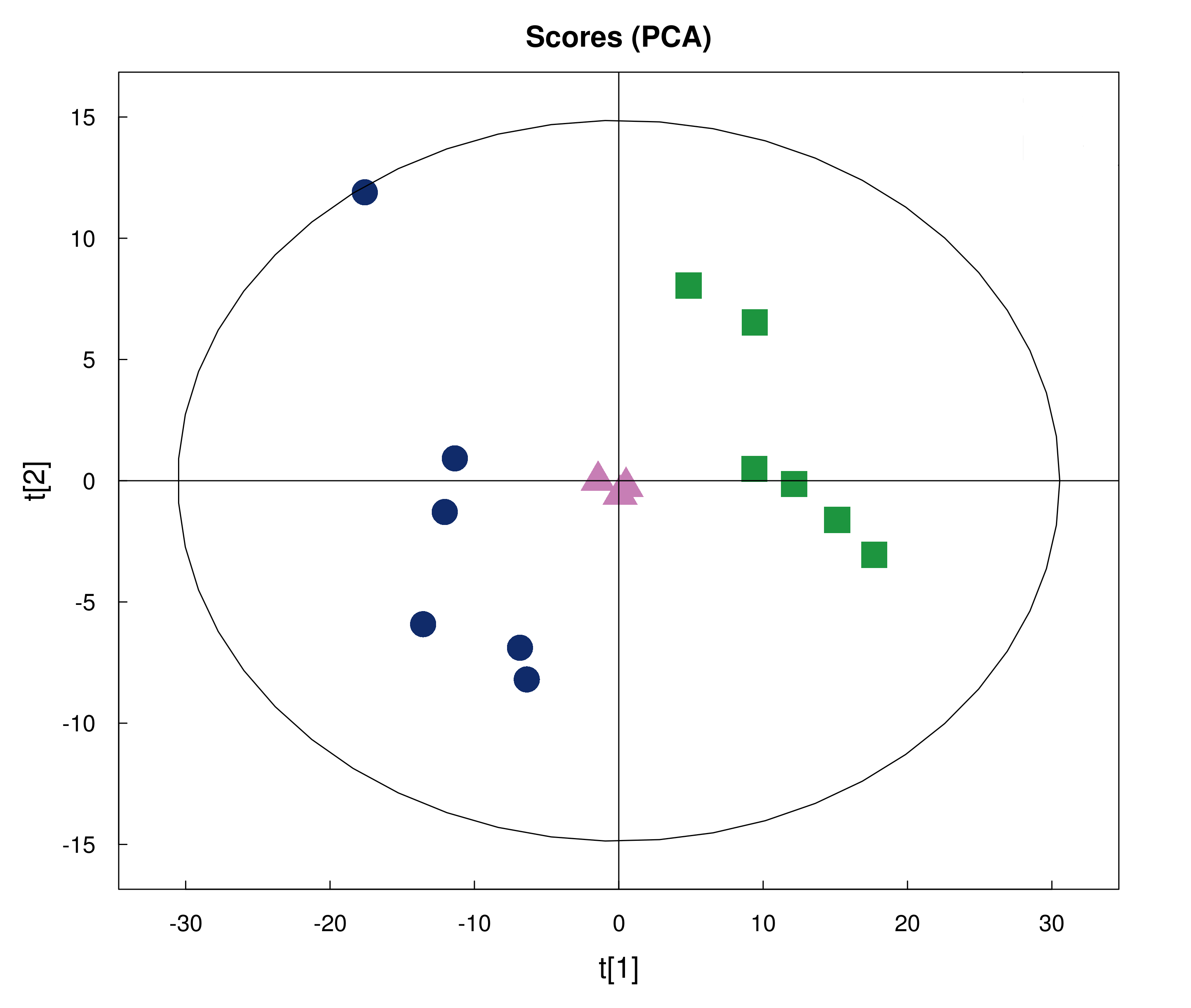

Supplement: Supplementary Figure 4 — Disease severity index (DSI) of honeysuckle after inoculation with E. lonicerae. After inoculation, the DSI of Honeysuckle continued to increase significantly from 14.2 at day 7 to 76.9 at day 56. The data were showed as mean ± SD (n = 5). Statistical significance are indicated by different letters (P < 0.05). [file Image_4.tif]

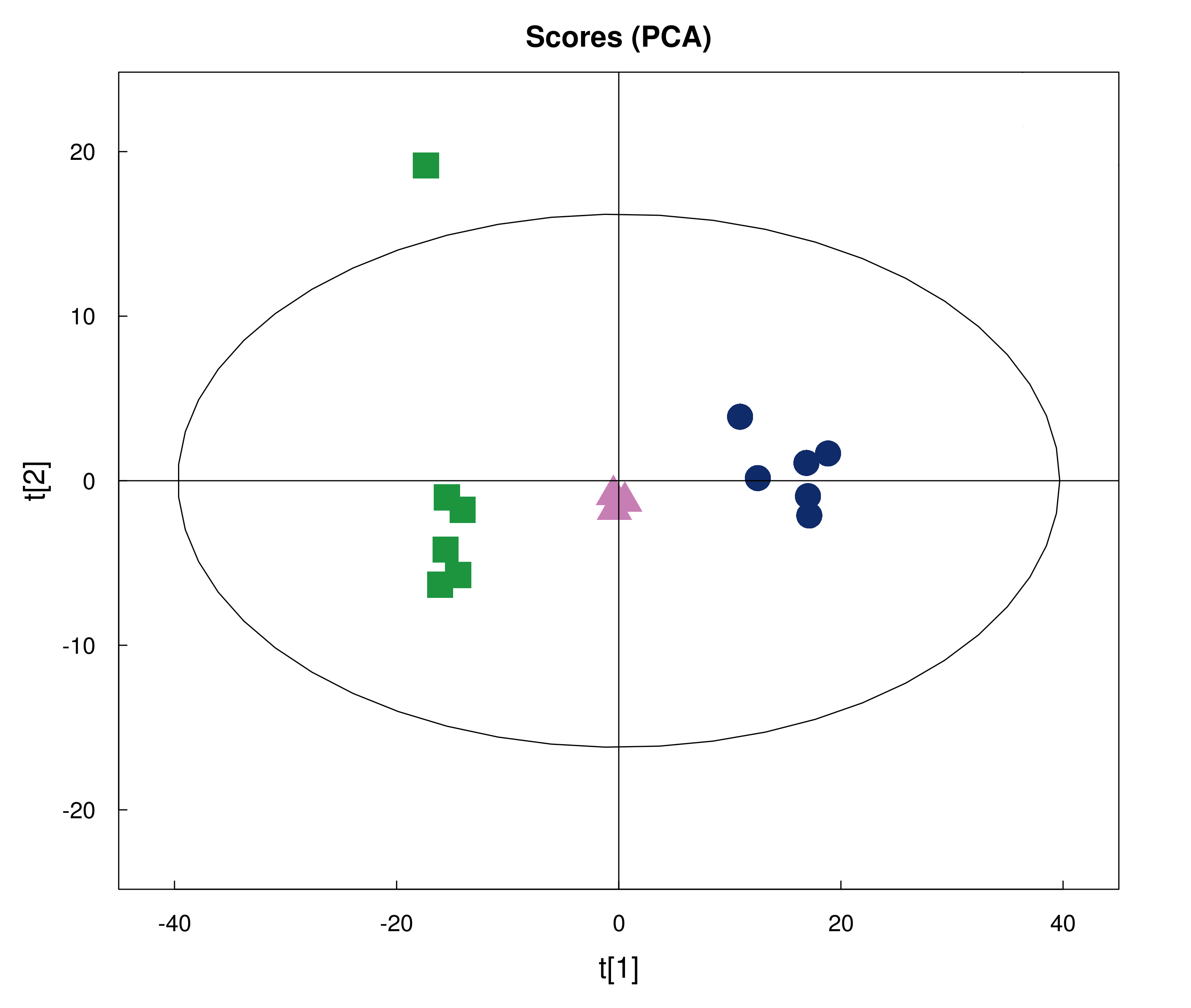

Supplement: Supplementary file 9 [file Image_5.tif]

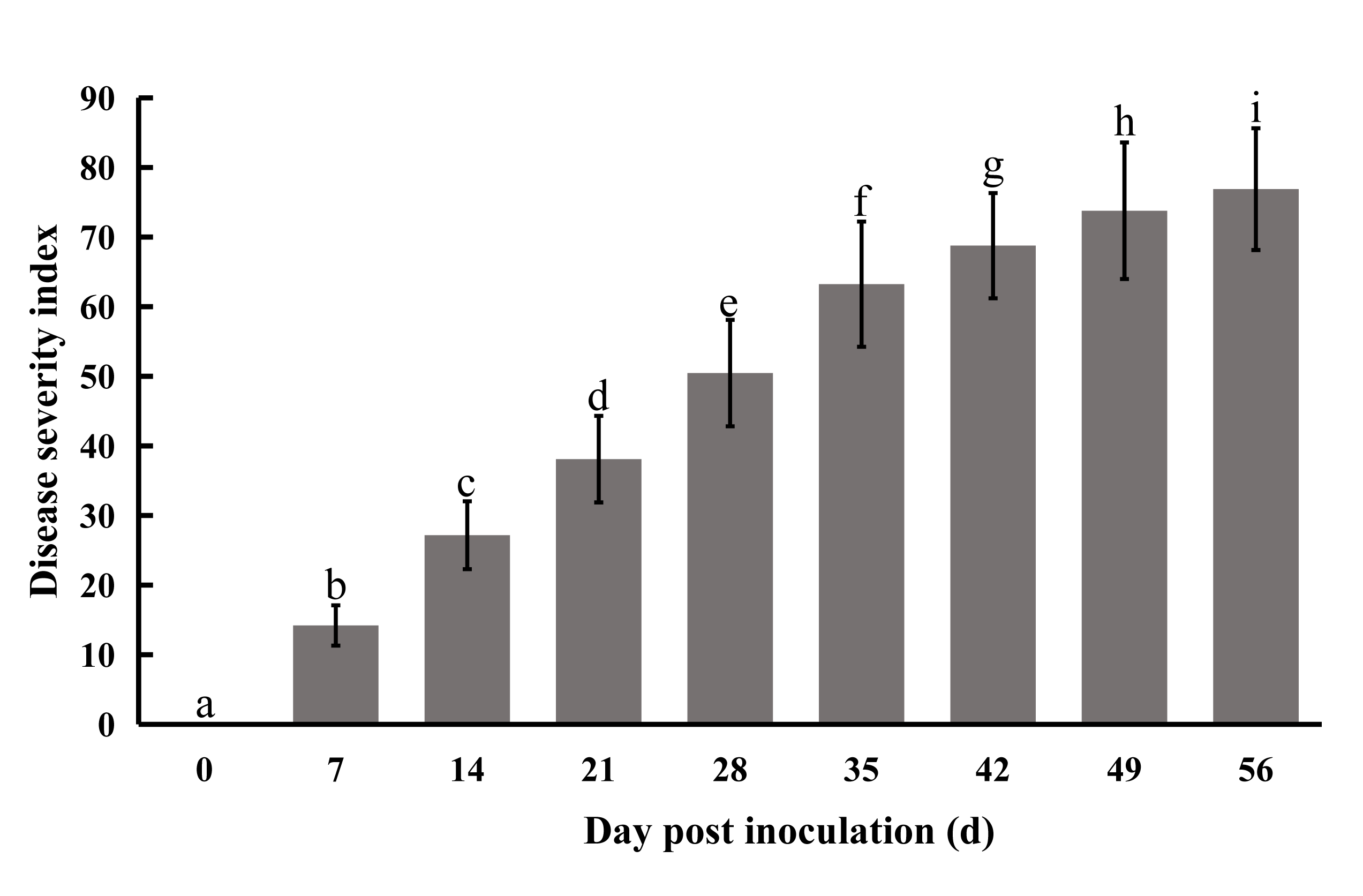

Supplement: Supplementary file 10 [file Image_6.tif]
