# Supplementary material for: Reduction of flavonoid content in honeysuckle via Erysiphe lonicerae-mediated inhibition of three essential genes in flavonoid biosynthesis pathways
Source: Front Plant Sci. 2024 Apr 16;15:1381368. doi: 10.3389/fpls.2024.1381368 (PMC11059088; doi:10.3389/fpls.2024.1381368)
Supplement: Supplementary Table 4 — Primers utilized in qpcr analysis. A detailed list of all primers used for quantitative PCR (qPCR) analysis in this study is provided. [file Table_4.docx]

| Gene | Primers |
| --- | --- |
| Actin-F | CGACTACGAGCAAGAACTTGA (Pu et al. 2020) |
| Actin-R | CGAACCACCACTAAGCACAA (Pu et al. 2020) |
| LjFNHO1-F | TCGATCGCACTCACATCGAG |
| LjFNHO1-R | GCTCTGCTTTGGCTTTCACC |
| LjFNGT1-F | TGCTATCATCCCCTCCACCA |
| LjFNGT1-R | CCCTTCTGGCAAGAGGCTTT |
| LjFNRT1-F | GCGCTGTTGTTTTGTACCCT |
| LjFNRT1-R | TGGAGGAATCCTGAGGGAGG |
| LjPAL1-F | GGACGGACAGTTACGGTGTT |
| LjPAL1-R | TGGAGGAGGGTGTTGATCCT |
| LjGNMO1-F | TGTTGACAGAGCCCGACTTC |
| LjGNMO1-R | GGGAATGTCGTAGCCACCAA |

Table S4 Primers used for qPCR analysis in this study.
